# Prediction of Intracranial Hypertension and Brain Tissue Hypoxia Utilizing High-Resolution Data from the BOOST-II Clinical Trial

**DOI:** 10.1089/neur.2022.0055

**Published:** 2022-10-27

**Authors:** Christos Lazaridis, Aswathy Ajith, Ali Mansour, David O. Okonkwo, Ramon Diaz-Arrastia, Anoop Mayampurath

**Affiliations:** ^1^ Departments of Neurology and Neurosurgery, University of Chicago Medical Center, University of Chicago, Chicago, Illinois, USA.; ^2^ Department of Computer Science, University of Chicago, Chicago, Illinois, USA.; ^3^ Department of Neurosurgery, University of Pittsburgh, Pittsburgh, Pennsylvania, USA.; ^4^ Department of Neurology, University of Pennsylvania Perelman School of Medicine, Philadelphia, Pennsylvania, USA.; ^5^ Department of Biostatistics and Medical Informatics, University of Wisconsin, Madison, Wisconsin, USA.

**Keywords:** brain hypoxia, intracranial hypertension, prediction, secondary brain injury, traumatic brain injury

## Abstract

The current approach to intracranial hypertension and brain tissue hypoxia is reactive, based on fixed thresholds. We used statistical machine learning on high-frequency intracranial pressure (ICP) and partial brain tissue oxygen tension (PbtO_2_) data obtained from the BOOST-II trial with the goal of constructing robust quantitative models to predict ICP/PbtO_2_ crises. We derived the following machine learning models: logistic regression (LR), elastic net, and random forest. We split the data set into 70–30% for training and testing and utilized a discrete-time survival analysis framework and 5-fold hyperparameter optimization strategy for all models. We compared model performances on discrimination between events and non-events of increased ICP or low PbtO_2_ with the area under the receiver operating characteristic (AUROC) curve. We further analyzed clinical utility through a decision curve analysis (DCA). When considering discrimination, the number of features, and interpretability, we identified the RF model that combined the most recent ICP reading, episode number, and longitudinal trends over the preceding 30 min as the best performing for predicting ICP crisis events within the next 30 min (AUC 0.78). For PbtO_2_, the LR model utilizing the most recent reading, episode number, and longitudinal trends over the preceding 30 min was the best performing (AUC, 0.84). The DCA showed clinical usefulness for wide risk of thresholds for both ICP and PbtO_2_ predictions. Acceptable alerting thresholds could range from 20% to 80% depending on a patient-specific assessment of the benefit-risk ratio of a given intervention in response to the alert.

## Introduction

Annually, >5.5 million people experience severe (sTBI) traumatic brain injury (TBI) worldwide.^1^ Outcomes have not substantially changed over the past 30 years with mortality of 30–40% and very limited breakthroughs after almost 200 randomized controlled trials (RCTs).^2,3^ There are few effective treatments for sTBI, and presently management is centered on the early evacuation of mass lesions and identification and treatment of secondary brain injury (SBI) that evolves in the hours and days after initial impact. Contemporary critical care of patients with sTBI aims to identify and manage SBI by monitoring of intracranial pressure (ICP), cerebral perfusion pressure (CPP), and partial brain tissue oxygen tension (PbtO_2_).^4^ This approach is recommended by the Brain Trauma Foundation guidelines and more recently by the Seattle International Severe TBI Consensus Conference.^5,6^ The therapeutic paradigm underlying these recommendations is a reactive one, where fixed, population-based treatment thresholds are observed and acted upon to alleviate SBI. However, by the time treatment is enacted, it may be too late. The ability to predict the onset of these “crisis” events would provide clinicians with valuable time to attempt aborting or manage these episodes more effectively, instead of merely reacting when thresholds are violated.^7^

Prediction efforts can be broadly divided into two approaches: 1) ICP forecasting, involving algorithms designed to predict future ICP values, and 2) ICP dose prediction, which involves algorithms aimed at the development of early warning systems of impending crisis events. Our work belongs to the latter category. A few studies have been published attempting to forecast future ICP values, or predict the onset of ICP crisis events, and one investigation has explored both ICP and PbtO_2_ dose predictions.^8–10^ In this article, we report on the performance and clinical usefulness of predictive models for intracranial hypertension and brain tissue hypoxia in high-frequency data obtained from the BOOST-II (Brain Oxygen Optimization in Severe Traumatic Brain Injury) phase II randomized trial.^11^ The objectives are 2-fold: 1) explore machine learning models utilizing high-frequency data and using a minimal set of features that can predict intracranial hypertension and brain tissue hypoxia insults as defined in BOOST-II; 2) show that this modeling is of clinical utility based on decision curve analysis (DCA).

## Methods

The BOOST II Data Set is the source of data for the present work, and the study has been approved by the University of Chicago (UChicago) institutional review board (IRB) under protocol IRB19-1847. The BOOST-II study was a two-arm, single-blind, prospective, randomized controlled multi-center phase II trial assessing safety and efficacy of a management protocol optimizing PbtO_2_ post-sTBI (ClinicalTrials.gov registration NCT: 00974259); 110 patients were randomized. After randomization, the control group (ICP only) was managed with a standard-of-care step-wise intervention strategy triggered by an ICP ≥20 mm Hg for >5 min. The intervention group (ICP + PbtO_2_) was medically managed with step-wise treatments to correct either an ICP increase or a reduction in PbtO_2_ (≤20 mm Hg, >5 min).

The study concluded that a treatment protocol guided by both ICP and PbtO_2_ reduces the duration of measured brain tissue hypoxia.^11^ The combined ICP/PbtO_2_ group was managed according to four types of events: A) no interventions; B) high ICP; C) low PbtO_2_; and D) high ICP + low PbtO_2_ (Supplementary Table S1 provides patient characteristics who experienced at least one ICP or PbtO_2_ event vs. patients who experienced no events). For ICP prediction, we investigated the succession of events from A or C to B or D; once this change was detected, subsequent observations were discarded (i.e., all B/D->B/D episodes were removed). For PbtO_2_, the succession used was A or B to C or D; once this change was detected, subsequent observations were discarded (i.e., all C/D->C/D removed). We constructed several sets of features for both outcomes. First, we used event number and the last recorded measurement. Then, we expanded to include trends (mean, median, standard deviation, minimum, maximum, difference between first and last recording, difference between most recent recordings, and area under the curve [AUC]) over the preceding 30 min.

Finally, we added frequency-domain measures (slope of power spectrum distribution, variance of power spectrum distribution, and approximate entropy). We derived the following machine learning models: logistic regression (LR), elastic net (EN), and random forest (RF). We split the data set into 70–30% for training and testing and utilized a discrete-time survival analysis framework and 5-fold hyperparameter optimization strategy for all models. Briefly, data within the training set were blocked into 30-min intervals where the last observations were chosen as representative of the block. Models were trained to predict the outcome within the next block. The test data set was not blocked when evaluating model performances. We compared model performances on discrimination between events and non-events of increased ICP or low PbtO_2_ with the area under the receiver operating characteristic (AUROC) curve. We further analyzed clinical utility or net benefit from making treatment decisions based on predictions through a DCA using a set of observations sampled from the test data set that had an equal proportion of outcomes.^12^

## Results

**FIG. 1.**
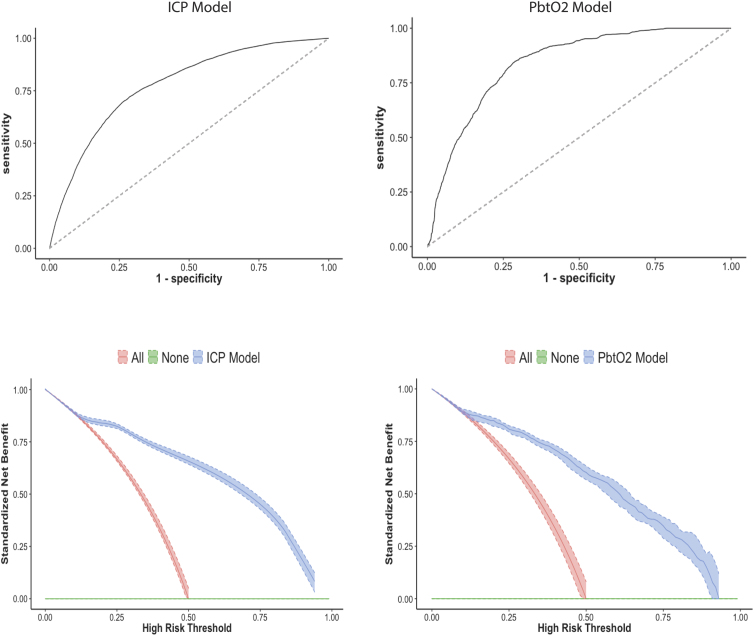
Upper panel: AUROC best model performances for ICP prediction, with the RF exhibiting best performance (AUROC, 0.78), whereas for PbtO_2_, the LR model was the best performing (AUROC, 0.84). Lower panel: decision curve analysis showing clinical usefulness for wide risk of thresholds for both ICP and PbtO_2_ predictions. AUROC, area under the receiver operating characteristic curve; ICP, intracranial pressure; RF, random forest; PbtO_2_, partial brain tissue oxygen tension; LR, logistic regression.

Figure 1 (upper panel) depicts the AUROC best model performances for ICP prediction, with the RF exhibiting best performance (AUROC, 0.78), whereas for PbtO_2_, the LR model was the best performing (AUROC, 0.84). Supplementary Tables S2 and S3 provide AUROCs for ICP and PbtO_2_ prediction within the next 30 min for the various models tested. In summary, the LR model that only used the most recent ICP reading and episode number predicted ICP crisis events within the next 30 min with good discrimination (AUC, 0.73; 95% confidence interval, 0.72–0.74). However, extending to an RF model improved performance (RF AUC 0.78 vs. LR AUC 0.73; *p* < 0.001). For PbtO_2_ crisis event prediction, the addition of longitudinal features to a feature set comprising the most recent PbtO_2_ reading and episode number did not improve performance of the LR model (AUC, 0.84 vs. 0.83). Extension to RF decreased performance (RF AUC 0.82 vs. LR AUC 0.84), indicating overfitting.

**FIG. 2.**
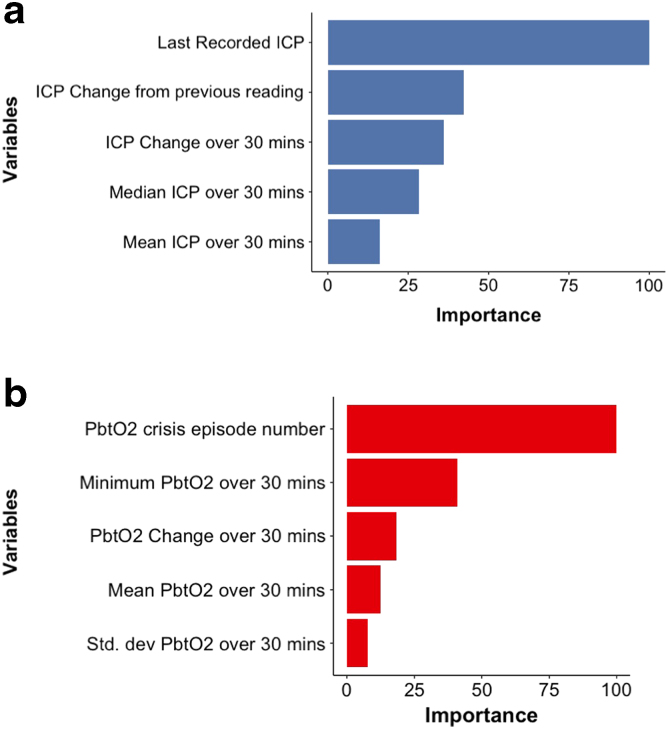
Feature variable importance plots. ICP, intracranial pressure; PbtO_2_, partial brain tissue oxygen tension.

Overall, when considering discrimination, number of features, and interpretability, we identified the RF model that combined the most recent ICP reading, episode number, and longitudinal trends over the preceding 30 min as the best performing model for predicting ICP crisis events within the next 30 min. An LR utilizing the most recent PbtO_2_ reading, episode number, and longitudinal trends over the preceding 30 min was the best performing model for predicting PbtO_2_ (see Fig. 2 for feature variable importance plots). The DCA showed clinical usefulness for a wide risk of thresholds for both ICP and PbtO_2_ predictions (Fig. 1, lower panel). Acceptable alerting thresholds could range from 20% to 80% depending on a patient-specific assessment of the benefit-risk ratio of a given intervention in response to the alert.

## Discussion

The approach underlying current management of ICP, CPP, and PbtO_2_ is based on mostly fixed, generic treatment thresholds as triggers for an escalating list of interventions. An important caveat is that by the time treatment is initiated, even if a return to desirable values is achieved, irreversible SBI may have occurred. This could partly explain the lack of effect, or indeed the negative clinical outcome, of a reactive management mode toward fixed values of ICP in guiding treatment.^13–15^ Combining statistical machine learning with clinical insight allows the construction of robust quantitative models to predict ICP/PbtO_2_ crises. Although previous work has been published on predicting ICP crises, these approaches used either low-resolution data, utilized hundreds of independent variables, or require hours-long epochs of monitoring to deliver predictions.^8–10,16^

Recently, Carra and colleagues undertook the validation testing of Gaussian processes (GPs)-based predictive modeling using the high-resolution CENTER-TBI data set.^10^ These algorithms demonstrated good intercenter robustness, with the model achieving an accuracy of 88%, sensitivity of 83%, and specificity of 91% in providing a 30-min forewarning of an ICP crisis (defined as an ICP >30 mm Hg lasting at least 10 consecutive min). However, using GPs, though promising with retrospective data, is computationally intensive and requires 4 h of input data to allow it to make a prediction. In contrast, we show here that it is possible to achieve reasonable predictive performance using few and clinically intuitive features, such as the most recent ICP/PbtO_2_ reading, episode number, and longitudinal trends over the preceding 30 min, to form models that are perhaps less prone to overfitting and more likely to generalize to clinical settings. The performance of these features is consistent with past work on ICP/PbtO_2_ predictive modeling from a single-center retrospective study of 817 sTBI patients based on prospectively collected physiological data.^9^

It should be noted that metrics of accuracy such as AUROC do not address the clinical value of a model, and, in fact, models with very different AUROCs can be comparable, or even models with higher AUROCs can sometimes lead to inferior clinical utility.^17^ For these reasons, we undertook DCA, as suggested by Steyerberg and colleagues.^12^ The decision curves demonstrate that the presented models can be of clinical utility, given that within a wide threshold range they provide higher net benefits than strategies of always treating (alert-all policy), and over current practice in which no warning exists (no alert policy). Setting an alerting threshold is a clinical decision, with acceptable thresholds ranging from 20% to 80% depending on a patient-specific assessment of the benefit-risk ratio of a given intervention in response to the alert; the riskier the intervention, the higher should the alerting threshold be.

These models require prospective validation to inform individualized prediction assessments in real time. Besides a prospective assessment of accuracy, real-time validation can provide mechanistic insights. A limitation of the presented purely data-driven predictive modeling is that it does not address the mechanisms behind predicted crises events. In order to design clinical management approaches, characterization of the mechanisms responsible for generating crises is further required. This approach may be novel in targeting SBI after TBI; nevertheless, it has been shown in other clinical environments that delivering alerts for predicted cardiorespiratory instability to providers leads to a marked decrease in both instability duration and the numbers of occurrences of serious instability episodes.^18,19^ Higher doses of intracranial hypertension, cerebral hypoperfusion, and brain tissue hypoxia have been associated with worse outcomes after sTBI.^9,11,20–23^ The ability to predict such events could enhance efforts to reduce the burden of these insults and, by extension, potentially improve functional outcomes.

## Conclusion

Combining statistical machine learning with clinical insight allows the construction of robust, clinically valuable quantitative models to predict ICP/PbtO_2_ crises. These models require prospective validation for their performance and in order to gain mechanistic insights. An accurate, automatic system of alarm delivery sets the stage for considering and testing a preemptive clinical algorithm for the prevention of crisis events. Such a clinical algorithm, if successful, could shift our treatment approaches from the current reactive mode to a preemptive one.

## Supplementary Material

Supplemental data

Supplemental data

Supplemental data

## The BOOST II Investigators

The BOOST II Investigators include: Ramon Diaz- Arrastia, MD, PhD, University of Pennsylvania Perelman School of Medicine, Philadelphia, PA; Nancy Temkin, PhD, University of Washington, Seattle, WA; Carol Moore, MA, CCRC, Southwestern Medical Center, Dallas, TX; Lori Shutter, MD, University of Pittsburgh School of Medicine, Pittsburgh, PA; Christopher Madden, MD, UT Southwestern Medical Center, Dallas, TX; Norberto Andaluz, MD, University of Cincinnati College of Medicine, Cincinnati, OH; David Okonkwo, MD, PhD, University of Pittsburgh School of Medicine, Pittsburgh, PA; Randall Chesnut, MD, University of Washington, Seattle, WA; Ross Bullock, MD, University of Miami, Miller School of Medicine, Miami, FL; John McGregor, MD, Ohio State University College of Medicine, Columbus, OH; Gerald Grant, MD, Stanford University, Stanford, CA; Mark Shapiro, MD, Duke University, Durham, NC; Michael Weaver, MD, Temple University, Philadelphia, PA; Peter LeRoux, MD, University of Rochester Medical Center, Rochester, NY; Jack Jallo, MD, PhD, Thomas Jefferson University, Philadelphia, PA.

## Source of the Data

The BOOST II Data Set was obtained by Data User Agreement between the University of Washington, on behalf of the BOOST II investigators, and the Data Recipient (Christos Lazaridis, UChicago).

## Abbreviations Used

AUC: area under the curve
AUROC: area under the receiver operating characteristic
CPP: cerebral perfusion pressure
DCA: decision curve analysis
EN: elastic net
GPs: Gaussian processes
ICP: intracranial pressure
IRB: institutional review board
LR: logistic regression
PbtO_2_: partial brain tissue oxygen tension
RF: random forest
SBI: secondary brain injury
sTBI: severe TBI
TBI: traumatic brain injury

## References

[1] Dewan MC, Rattani A, Gupta S, et al. Estimating the global incidence of traumatic brain injury. J Neurosurg 2018. doi: 10.3171/2017.10.JNS17352 29701556

[2] Bragge P, Synnot A, Maas AI, et al. A State-of-the-science overview of randomized controlled trials evaluating acute management of moderate-to-severe traumatic brain injury. J Neurotrauma 2016;33(16):1461–1478. 26711675 10.1089/neu.2015.4233 PMC5003006

[3] Bowman K, Matney C, Berwick DM. Improving traumatic brain injury care and research: a report from the National Academies of Sciences, Engineering, and Medicine. JAMA 2022;327(5):419–420. 35103760 10.1001/jama.2022.0089

[4] Lazaridis C, Robertson CS. The role of multimodal invasive monitoring in acute traumatic brain injury. Neurosurg Clin N Am 2016;27(4):509–517. 27637400 10.1016/j.nec.2016.05.010

[5] Carney N, Totten AM, O'Reilly C, et al. Guidelines for the Management of Severe Traumatic Brain Injury, Fourth Edition. Neurosurgery 2017;80(1):6–15. 27654000 10.1227/NEU.0000000000001432

[6] Chesnut R, Aguilera S, Buki A, et al. A management algorithm for adult patients with both brain oxygen and intracranial pressure monitoring: the Seattle International Severe Traumatic Brain Injury Consensus Conference (SIBICC). Intensive Care Med 2020;46(5):919–929. 31965267 10.1007/s00134-019-05900-x PMC7210240

[7] Lazaridis C, Rusin CG, Robertson CS. Secondary brain injury: predicting and preventing insults. Neuropharmacology 2019 Feb;145(Pt B):145–152. 10.1016/j.neuropharm.2018.06.005 29885419

[8] Güiza F, Depreitere B, Piper I, et al. Novel methods to predict increased intracranial pressure during intensive care and long-term neurologic outcome after traumatic brain injury: development and validation in a multicenter dataset. Crit Care Med 2013;41(2):554–564. 23263587 10.1097/CCM.0b013e3182742d0a

[9] Myers RB, Lazaridis C, Jermaine CM, et al. Predicting intracranial pressure and brain tissue oxygen crises in patients with severe traumatic brain injury. Crit Care Med 2016;44(9):1754–1761. 27315192 10.1097/CCM.0000000000001838 PMC7083460

[10] Carra G, Güiza F, Depreitere B, et al. Prediction model for intracranial hypertension demonstrates robust performance during external validation on the CENTER-TBI dataset. Intensive Care Med 2021;47(1):124–126. 33001234 10.1007/s00134-020-06247-4

[11] Okonkwo DO, Shutter LA, Moore C, et al. Brain oxygen optimization in severe traumatic brain injury phase-II: a phase II randomized trial. Crit Care Med 2017;45(11):1907–1914. 29028696 10.1097/CCM.0000000000002619 PMC5679063

[12] Steyerberg EW, Vickers AJ, Cook NR, et al. Assessing the performance of prediction models: a framework for traditional and novel measures. Epidemiology 2010;21(1):128–138. 20010215 10.1097/EDE.0b013e3181c30fb2 PMC3575184

[13] Shafi S, Diaz-Arrastia R, Madden C, et al. Intracranial pressure monitoring in brain-injured patients is associated with worsening of survival. J Trauma 2008;64(2):335–340. 18301195 10.1097/TA.0b013e31815dd017

[14] Chesnut RM, Temkin N, Carney N, et al. A trial of intracranial-pressure monitoring in traumatic brain injury. N Engl J Med 2012;367(26):2471–2481. 23234472 10.1056/NEJMoa1207363 PMC3565432

[15] Bennett TD, DeWitt PE, Greene TH, et al. Functional outcome after intracranial pressure monitoring for children with severe traumatic brain injury. JAMA Pediatr 2017;171(10):965–971. 28846763 10.1001/jamapediatrics.2017.2127 PMC5710627

[16] Güiza F, Depreitere B, Piper I, et al. Early detection of increased intracranial pressure episodes in traumatic brain injury: external validation in an adult and in a pediatric cohort. Crit Care Med 2017;45(3):e316–e320. 10.1097/CCM.0000000000002080 27632671

[17] Vickers AJ, Elkin EB. Decision curve analysis: a novel method for evaluating prediction models. Med Decis Making 2006;26(6):565–574. 17099194 10.1177/0272989X06295361 PMC2577036

[18] Hravnak M, Devita MA, Clontz A, et al. Cardiorespiratory instability before and after implementing an integrated monitoring system. Crit Care Med 2011;39(1):65–72. 20935559 10.1097/CCM.0b013e3181fb7b1c PMC3673290

[19] Wijnberge M, Geerts BF, Hol L, et al. Effect of a machine learning-derived early warning system for intraoperative hypotension vs standard care on depth and duration of intraoperative hypotension during elective noncardiac surgery: the HYPE Randomized Clinical Trial. JAMA 2020;323(11):1052–1060. 32065827 10.1001/jama.2020.0592 PMC7078808

[20] Lazaridis C, DeSantis SM, Smielewski P, et al. Patient-specific thresholds of intracranial pressure in severe traumatic brain injury. J Neurosurg 2014;120(4):893–900. 24506248 10.3171/2014.1.JNS131292

[21] Güiza F, Meyfroidt G, Piper I, et al. Cerebral perfusion pressure insults and associations with outcome in adult traumatic brain injury. J Neurotrauma 2017;34(16):2425–2431. 28385097 10.1089/neu.2016.4807 PMC5563857

[22] Åkerlund CA, Donnelly J, Zeiler FA, et al. Impact of duration and magnitude of raised intracranial pressure on outcome after severe traumatic brain injury: a CENTER-TBI high-resolution group study. PLoS One 2020;15(12):e0243427. 33315872 10.1371/journal.pone.0243427 PMC7735618

[23] Zeiler FA, Ercole A, Cabeleira M, et al. Patient-specific ICP epidemiologic thresholds in adult traumatic brain injury: a CENTER-TBI validation study. J Neurosurg Anesthesiol 2021;33(1):28–38. 31219937 10.1097/ANA.0000000000000616

