# Supplementary material for: Prediction of Intracranial Hypertension and Brain Tissue Hypoxia Utilizing High-Resolution Data from the BOOST-II Clinical Trial
Source: Neurotrauma Rep. 2022 Oct 27;3(1):473–8. doi: 10.1089/neur.2022.0055 (PMC9622207; doi:10.1089/neur.2022.0055)
Supplement: Supplemental data [file Supp_TableS1.docx]

|  | Patients who experienced at least one ICP event  (n=80) | Patients who did not experience ICP events  (n=34) | Patients who experienced at least one PbtO2 event  (n=40) | Patients who did not experience  PbtO2 events  (n=74) |
| --- | --- | --- | --- | --- |
| Sex, n (%) |  |  |  |  |
| Male | 64 (80) | 26 (77) | 35 (88) | 55 (74) |
| Female | 16 (20) | 8 (23) | 5 (12) | 19 (26) |
| Race, n (%) |  |  |  |  |
| Black | 9 (11) | 3 (9) | 3 (8) | 9 (12) |
| White | 67 (84) | 29 (85) | 34 (85) | 62 (84) |
| Other | 4 (5) | 2 (6) | 3 (8) | 3 (4) |
| Hispanic, n (%) | 18 (23) | 2 (6) |  |  |
| Number of events, n (%) |  |  |  |  |
| 0 | 0 (0) | - | 0 (0) | - |
| 1 | 14 (18) |  | 10 (25) |  |
| 2 | 5 (6) |  | 7 (17) |  |
| 3+ | 61 (76) |  | 23 (58) |  |

**Table 1**: Characteristics of patients who experience at least one ICP or PbtO2 event in comparison with patients who did not experience ICP or PbtO2 events respectively
