# Supplementary material for: Prediction of Intracranial Hypertension and Brain Tissue Hypoxia Utilizing High-Resolution Data from the BOOST-II Clinical Trial
Source: Neurotrauma Rep. 2022 Oct 27;3(1):473–8. doi: 10.1089/neur.2022.0055 (PMC9622207; doi:10.1089/neur.2022.0055)
Supplement: Supplemental data [file Supp_TableS2.docx]

**Table 2:** AUROC metrics for predicting ICP events within the next 30 mins for various features and machine learning models.

| Feature set | Logistic Regression Model AUROC (95% CI) | Elastic Net Model AUROC (95% CI) | Random Forest Model AUROC (95% CI) |
| --- | --- | --- | --- |
| Most recent ICP observation + episode number | 0.729 (0.722-0.737) | - | - |
| + ICP trends in prior 30 mins | 0.728 (0.720-0.735) | 0.779 (0.773-0.785) | 0.777 (0.771-0.783) |
| + ICP frequency-based features from prior 30 mins | 0.728 (0.720-0.735) | 0.773 (0.767-0.779) | 0.784 (0.779-0.791) |
