# Supplementary material for: Prediction of Intracranial Hypertension and Brain Tissue Hypoxia Utilizing High-Resolution Data from the BOOST-II Clinical Trial
Source: Neurotrauma Rep. 2022 Oct 27;3(1):473–8. doi: 10.1089/neur.2022.0055 (PMC9622207; doi:10.1089/neur.2022.0055)
Supplement: Supplemental data [file Supp_TableS3.docx]

**Table 3:** AUROC metrics for predicting PbtO2 events within the next 30 mins for various features and machine learning models.

| Feature set | Logistic Regression Model AUROC (95% CI) | Elastic Net Model AUROC (95% CI) | Random Forest Model AUROC (95% CI) |
| --- | --- | --- | --- |
| Most recent PbtO2 observations + episode number | 0.835 (0.827-0.843) | - | - |
| + PbtO2 trends in prior 30 mins | 0.841 (0.833-0.849) | 0.837 (0.828-0.845) | 0.822 (0.891-0.835) |
| + PbtO2 frequency-based features from prior 30 mins | 0.838 (0.829-0.847) | 0.838 (0.829-0.846) | 0.804 (0.791-0.818) |
